# Building an outpatient telemedicine care pilot using Scrum-like framework within a medical residency program

**DOI:** 10.6061/clinics/2021/e2795

**Published:** 2021-06-07

**Authors:** Kaio Jia Bin, Natalia Higa, Jéssica Helena da Silva, Daniele Abud Quagliano, Rosemeire Keiko Hangai, Vilson Cobello-Júnior, Antonio José Rodrigues Pereira, Luiz Augusto Carneiro-D'Albuquerque, Flair José Carrilho, Chao Lung Wen, Suzane Kioko Ono

**Affiliations:** I Programa de Estudos Avancados em Administracao Hospitalar e de Sistemas de Saude (PROAHSA), Hospital das Clinicas HCFMUSP, Faculdade de Medicina, Universidade de Sao Paulo, Sao Paulo, SP, BR; II Hospital das Clinicas HCFMUSP, Faculdade de Medicina, Universidade de Sao Paulo, Sao Paulo, SP, BR; III Nucleo Especializado em Tecnologia da Informacao (NETI), Hospital das Clinicas HCFMUSP, Faculdade de Medicina, Universidade de Sao Paulo, Sao Paulo, SP, BR; IV Superintendencia, Hospital das Clinicas HCFMUSP, Faculdade de Medicina, Universidade de Sao Paulo, Sao Paulo, SP, BR; V Departamento de Gastroenterologia, Hospital das Clinicas HCFMUSP, Faculdade de Medicina, Universidade de Sao Paulo, Sao Paulo, SP, BR; VI Telemedicina, Faculdade de Medicina FMUSP, Universidade de Sao Paulo, Sao Paulo, SP, BR

**Keywords:** Scrum, Telemedicine, Agile Methodology, Telehealth, COVID-19

## Abstract

**OBJECTIVES:**

A good health care does not only depend on good medical practice, but also needs great management of its resources, which are generally short. In this sense, PROAHSA has been training new health managers since 1972. With the arrival of the COVID-19 pandemic, it was clear that medicine will go through a new phase, where telehealth will be present in this “Improved Normal”. This report is about how a pilot teleconsultation study was carried out for HCFMUSP patients through the Scrum-like framework. It is to deploy a pilot of remote assistance involving a doctor and a patient in the Ambulatory of Hepatology and Liver Transplantation of HCFMUSP.

**METHODS:**

We applied the Scrum-like framework to carry out this work with an interdisciplinary multifunctionality team.

**RESULTS:**

A full telemedicine service flow was implemented within eight weeks using existing infrastructure and resources implementing the Scrum methodology. Twenty-three teleconsultations were scheduled and eight guides built.

**CONCLUSION:**

Scrum framework has a great potential to improve the training of students and to conclude pilot projects.

## INTRODUCTION

The millennial battle between man and microorganisms gained another chapter in 2020. With the COVID-19 pandemic we achieved an “Improved Normal,” where the field of telemedicine evolved importantly with no turning back.

Currently the Hospital das Clínicas of the University of São Paulo School of Medicine (HCFMUSP), occupies a total area of 600 thousand square meters with approximately 2,400 beds distributed among its eight specialized institutes and one auxiliary hospital.

Among the units we have the “Prédio dos Ambulatórios” (PAMB), a building specialized in outpatient care and day hospital, in addition to areas of support and therapeutic diagnosis. It has more than 30 clinical and surgical specialties and performs around 1.4 million outpatient consultations each year.

This study was carried out at the Clinical Gastroenterology and Hepatology Division and Liver Transplantation and Digestive System Organs Division of the HCFMUSP, which performs approximately 20,000 outpatient consultations per year. It has a team of 23 assistant doctors and 25 resident doctors.

In mid-March 2020, outpatient consultations and procedures were canceled. In addition, the “Programa HC em Casa” (HC at Home program), a new system for rescheduling appointments, inter-consultations, and future outpatient procedures (laboratory / image exams and prescriptions prevalidation), was launched in April 2020.

Outpatient consultations were resumed in August 2020. But the HCFMUSP is still concerned about patient safety, and is looking for alternatives for outpatient care. Due to the pandemic caused by COVID-19, the use of telemedicine was allowed for patient care, according to Ministry Ordinance No. 467 of 03/20/2020.

Due to the urgency of the COVID-19 pandemic, a quick adaptation was needed to provide care to outpatients in social isolation. PROAHSA, in partnership with the Clinical Gastroenterology and Hepatology Division of HCFMUSP, set up a pilot of attendance by video conference, utilizing Scrum framework.

### Telemedicine

Medicine is the set of scientific and technical knowledge for the prevention, treatment, and cure of diseases, disorders, and injuries according to the Michaelis dictionary. The prefix “tele,” of Greek origin, means “distance.” Thus, in a very succinct way we can say that telemedicine is medicine performed at a distance, where individuals are not in the same place.

In considering the practice of prevention as an act of medicine, the use of smoke to inform about the advance of the bubonic plague in Europe in the Middle Ages, can be considered one of the first examples of telemedicine (1). With the development of information and communication technologies, such as telephone, radio, television, and cell phones, telemedicine spread throughout society, and mainly with the use of personal electronic devices.

According to the World Health Organization (WHO), telemedicine is “the provision of services related to health care, in cases where distance is a critical factor: such services are provided by health professionals, using information and communication technologies for the exchange of valid information for the diagnosis, prevention, and treatment of diseases and the continuous education of health care providers, as well as for research and evaluation purposes; all in the interest of improving the health of people and their communities” (2).

### Scrum Framework

Scrum is not a process, technique, or method, it is a framework where people can treat and solve complex problems, to deliver products with the highest possible value (3). Currently, 60% of agile methodology projects employ the Scrum framework as project management (4).

Briefly, this framework has three main components: The Team, the Artifacts, and the Events.

### Objective

Deploy a pilot of remote assistance involving a doctor and a patient in the Ambulatory of Hepatology and Liver Transplantation of the HCFMUSP, in a sustainable way using existing resources, with a detailed description of the pre and post teleconsultation activity’s workflows, as well as the consent terms for the patients.

## MATERIALS AND METHODS

Scrum concept was considered for this project (3). Roles, artifacts, and events were modified according to our experience, as follows:

### Scrum Team Roles:

- Product Owner: Medical Chief of the Ambulatory care clinic of Clinical Gastroenterology and Hepatology Division of HCFMUSP
- Scrum Master: Deputy Director of PROAHSA
- Developers: a resident doctor of PROAHSA and two Hospital Managers of the Hepatology and Liver Transplantation Ambulatory

### Events:

- The Sprint: each Sprint lasted one week.
- Daily Scrum: held through the exchange of messages by cell phone between the entire Scrum team.
- Sprint Planning: adapted to the reality of PROAHSA, weekly monitoring was carried out between the Deputy Director and Resident Doctor to review actions from the previous week, and planning for the following week.
- Sprint Review: adapted to the reality of PROAHSA, a final presentation was made to the Medical Chief of the Clinic and the entire Scrum team at the end of four weeks.
- Sprint Retrospective: adapted to the reality of PROAHSA, a feedback meeting was held between the Deputy Director and Resident Doctor every week on Friday.

### Artifact (Product Backlog)

1. Prior appointment with the patient
2. Reception of the patient before the medical consultation
3. Doctor's video conference with the patient
4. Post-patient consultation
5. Return schedule
6. Scheduling exams
7. Scheduling medicines with home delivery

### Criteria for Participation in the Teleconsultation:

- A clinically stable patient with no need for a physical examination during teleconsultation.
- A patient having a cell phone and / or computer with a camera and internet access.
- A patient familiar with video calling.
- A patient agreeing to the teleconsultation.
- Not being the first outpatient appointment.
- A patient having performed a video call test while scheduling.

### Technology

Institutional Google Meet (HIPAA compliant) was used for a video conference between the doctor and patient.

Pre-selected patients by the Medical Chief of the Ambulatory of Gastroenterology were contacted through a cell phone call or WhatsApp message to make the appointment.

One WhatsApp account and an institutional email account were created for the patient’s schedule. An event in the email schedule function was created with the consultation date and time, inviting the patient informed by email during the call. We still check if the patient receives the invitation by email.

During the consultation we used a webcam for image and audio transmission.

Post-consultation activities, such as exam requests and prescriptions are sent to the local front desk, where they will be scheduled, and instructions are sent by mail to patients.

### Planning

The planning and follow-up were based on the Scrum framework, to provide transparency, inspection, and adaptation to the project. Even more so in the context of the pandemic, which called for a speedy pilot project with the use of medical assistance by video call to assist and help patients with teleconsultation.

### Scheduling

The scheduling was performed by the Developers team, once a week, always on a Monday morning within 4-hour periods, using phone calls.

The candidates’ list for the pilot project is part of the Product Owner's return schedule.

### Teleconsultation

The teleconsultation was performed by the Product Owner, once a week, always on Thursday morning.

It was performed via Google Meet^®^ using an institutional mail created for this pilot to invite the patient’s personal mail for the videoconference.

### Pre and Post Teleconsultation Activities and Other Documents

Standard operating procedure (SOP) and the Free and Informed Consent Form (FICF) will be built using the existing workflow for non-remote assistance as benchmarking.

## RESULTS

During the pilot project, in the eight-week period (between June and August 2020), documents were compiled to guide the medical assistance team, the administrative team, and the patients. Among the documents constructed for the assistance team there were:

1. FICF - before the teleconsultation
2. SOP for teleconsultation

In addition, an approach to the dissemination and training on telemedicine for the medical assistance team was also carried out by offering an online course on data security and telemedicine.

To guide the administrative team, the following documents were constructed:

1. Post-consultation SOP
2. Standardization of the teleconsultation confirmation email and date of the outpatient return visit
3. Scheduling SOP

For the patient, the following were provided:

1. Google Meet (platform used for video calling with the doctor) Access Instruction guide
2. Patient guidance on medical prescriptions, complementary exams, and scheduling outpatient visits

This pilot project had three main points: scheduling, teleconsultation, and post-consultation, which were standardized and structured with the construction of the documents mentioned above.

The scheduling and post-consultation processes were carried out by telephone or WhatsApp together with the use of the documents “Criteria for participation in teleconsultation,” “Scheduling SOP,” “Post-consultation SOP,” “Standardization of the teleconsultation confirmation email and date of the outpatient return visit,” and “Patient guidance on medical prescription, complementary exams, and scheduling of outpatient return.”

In addition, the teleconsultation platform was tested, and the “Google Meet Access Instructions (the platform used for video calls with the doctor)” were sent to the patient.

The teleconsultation was based on the FICF and Teleconsultation SOP.

During the eight weeks for this activity, 28 scheduling attempts were made. The activity of scheduling was performed once a week, lasting four hours each. There was a conversion of 82.1% of these attempts for scheduling, which corresponds to 23 scheduled teleconsultations.

Each attempt for scheduling took about nine minutes for explaining to the patients the schedule and how to use the Google Meets^®^.

There were five unsuccessful attempts to schedule, two did not answer a cell phone call, two had not performed complementary exams needed for the teleconsultation, and one refused to be treated by telemedicine.

The teleconsultations of the pilot project were once a week, in the morning. Altogether 14 teleconsultations were carried out and six teleconsultations were scheduled. There were three unscheduled teleconsultations because the patient changed his mind and changed the preference for face-to-face consultation or needed to reschedule.

The average time to perform the schedule was 21 minutes, which included the explanation of the pilot project, verification of inclusion criteria, and the test on the video call platform.

The average time for teleconsultation was 15 minutes, varying from the application of FICF confirmation to the time of issuing the prescription and requesting additional tests, when necessary.

## DISCUSSION

Scrum framework is immensely popular among large software development companies, because its light, flexible, and transparent structure facilitates much in the development management of numerous technology projects. According to the 14^th^ Annual State of Agile Report (4), Scrum and its related variants are used by 58% of the respondents’ organizations.

Along with the multifunctionality characteristic, the Scrum methodology has been increasingly used to form interdisciplinary teams to carry out projects involving assistance and information technology, such as creating software that improves patient care (5-7), training of health professionals (8,9), or improving the efficiency of using the Electronic Health Record (10,11), and for managing science projects (12).

The present report is a description of how PROAHSA adapted this methodology in the training of its students, absorbing its concepts of flexibility, agility, and transparency.

Based on the results and experience during the internship period, the importance of using Scrum is noted, a tool that helps in solving complex problems and promotes the delivery of viable products.

Periodic meetings and quick decisions to adapt to the needs of outpatients were essential to ensure adequate care and delivery of prescriptions and exams requests, without any patient’s impairment.

At each delivery, attempts were made to improve the flows and processes of the teleconsultation and also to prevent the possible risks imagined and encountered.

For all this to happen, the values brought by Scrum such as leadership, focus on work, team commitment, and respect were essential points for the execution of the pilot project and construction of the best delivery for the assistance team, local management team, and patients.

Large projects with many doubts about feasibility, such as this about telemedicine, could be put into practice without jeopardizing the normal course of the routines.

Within a few weeks, at no additional cost, guides and flowcharts could be drawn up. And so, a viable model can be brought to a greater discussion on the topic, without the need for immediate investment or making decisions that dramatically change the processes of those involved.

The assembled pilot service may not meet all expectations. However, it opened up the possibility of discussing its relevance and continuity.

And in times of crisis like a pandemic, quick solutions are needed for the benefit of patients. And agile methodologies like Scrum showed its effectiveness in this work by allowing the completion of a pilot from its planning to its execution in just eight weeks.

### Limitations

Difficulty with cultural change, such as scheduling an appointment on Google Meet, was overcome by centralizing this activity in the role of the medical resident. However, for mass implementation, administrative staff will need to receive adequate training and support.

This Scrum-like framework model did not include mentoring of local employees, because the focus of PROAHSA is the resident student’s training.

Owing to the agility dynamics of the methodology, where a large volume of actions and knowledge are generated in a short time, we noticed that during the execution of this project, we often prioritized “doing” and not “writing,” focusing emphasis on empiricism and lacking on documentation (13). As mentioned by Boehm (14), “Agile methods derive much of their agility by relying on the tacit knowledge embodied in the team, rather than writing the knowledge down in plans.” We encountered during the project that all preparation and planning were scattered in exchanges of electronic messages. An additional effort was necessary to gather the required information to describe the entire process, and one of the reasons for this publication is to document precisely the applied methodology for future works.

About support, an IT team was needed, we have full cooperation from the local team, and also from the corporate information technology and clinical engineering team that provided us with all the necessary infrastructure and equipment, such as computer, video camera, and wireless internet network. Without all this support, this work would not have been completed.

About Scrum’s scalability within complex HCFMUSP, we recommend more projects being developed using this methodology and a new evaluation model be created for this method.

### Cost

The total cost of equipment involved was low, we set up a webcam on the existing computer in the ambulatory's research room, with an additional monitor. That is, the only equipment needed were a webcam and a 21” Monitor.

## CONCLUSION

Scrum framework showed efficiency for the implementation of a telemedicine project in a complex outpatient environment.

Patient remote assistance was successfully performed at the Ambulatory of Hepatology and Liver Transplantation for the HCFMUSP with low investment using its own resources through electronic facilities.

SOP, FICF, guidelines, and workflows were built within eight weeks while scheduling and teleconsultation were carried out.

Time and money have been saved. And now, with a fully functioning pilot, improvements and expansion can be evaluated.

## AUTHOR CONTRIBUTIONS

Bin KJ, Higa N, Silva J, Quagliano DA, and Ono SK were responsible for the study conception/design, data acquisition, analysis and interpretation, manuscript drafting, review, and final approval. Hangai RK, Cobello-Junior V, Pereira AJR, Carneiro D’Albuquerque LA, Carrilho FJ, and Wen CL were responsible for the data analysis and interpretation, manuscript drafting, review, and final approval.
